# Do serum homocysteine levels affect female sexual function in reproductive-age women: a cross-sectional study

**DOI:** 10.1590/1806-9282.20260293

**Published:** 2026-07-20

**Authors:** Bilge Dogan Taymur, Güldeniz Toklucu, Sümeyye Yayla

**Affiliations:** 1 University of Health Sciences, Sehit Prof. Dr. Ilhan Varank Sancaktepe Training and Research Hospital, Department of Obstetrics and Gynecology – Istanbul, Turkey.

**Keywords:** Homocysteine, Sexual dysfunction, Vitamin B12, Folic acid

## Abstract

**OBJECTIVE:**

The aim of this study was to investigate the association between serum homocysteine levels and female sexual function in reproductive-age women presenting with sexual dysfunction complaints and to evaluate relationships with vitamin B12 and folic acid levels.

**METHODS:**

This cross-sectional study included 114 sexually active women aged 18–45 years presenting with sexual dysfunction complaints. Female sexual function was assessed using the Female Sexual Function Index. Fasting serum homocysteine, vitamin B12, and folic acid levels were measured. Correlation analyses, group comparisons, and multivariable linear regression were performed. A priori sample size calculation indicated that at least 84 participants were required to detect a moderate correlation (ρ=0.30) with 80% power.

**RESULTS:**

Female Sexual Function Index scores were available for 111 participants and homocysteine levels for 99 participants. The mean Female Sexual Function Index total score was 24.69±4.34, below the established cut-off for sexual dysfunction. Serum homocysteine levels were not associated with Female Sexual Function Index total or domain scores (ρ=-0.035, p=0.726). No significant differences were observed between homocysteine groups defined by ≤10 and >10 μmol/L or ≤15 and >15 μmol/L. Age showed a moderate negative correlation with the desire domain (ρ=-0.362, p<0.001). In multivariable regression, homocysteine, vitamin B12, and folic acid were not independent predictors of Female Sexual Function Index total score.

**CONCLUSION:**

In this symptomatic population, homocysteine levels were not associated with female sexual function. These findings should be interpreted cautiously due to the lack of a control group. Further large-scale, population-based studies are needed.

## INTRODUCTION

Female sexual dysfunction is a multifactorial condition influenced by biological, hormonal, psychological, and relational factors^1-8^. The Female Sexual Function Index (FSFI) is a validated multidimensional instrument widely used to evaluate female sexual function across six domains^1-3^. Homocysteine is a sulfur-containing amino acid involved in methionine metabolism. Elevated serum homocysteine has been associated with endothelial dysfunction, oxidative stress, and impaired nitric oxide bioavailability, contributing to vascular disease^9^. In males, hyperhomocysteinemia has been associated with erectile dysfunction and vascular impairment^9-11^. Population-based data suggest sex-specific differences in this relationship. Yang et al. demonstrated that increased homocysteine levels were associated with reduced sexual frequency in men but not in women^9^. These findings suggest that vascular biomarkers may affect male and female sexual function differently.

Despite the recognized vascular component of genital arousal, studies examining serum homocysteine and female sexual function are scarce. The current study sought to assess the correlation between serum homocysteine concentrations and FSFI total and domain scores in women of reproductive age, as well as to investigate the possible influences of vitamin B12 and folic acid levels.

## METHODS

### Study design and participants

This cross-sectional study was conducted at a tertiary gynecology outpatient clinic between November 2023 and February 2024. Sexually active women aged 18–45 years presenting with sexual dysfunction complaints were consecutively recruited.

Exclusion criteria included the following: pregnancy, thyroid disease, diabetes, polycystic ovary syndrome, use of antidepressants or oral contraceptives, severe systemic diseases, endocrine disorders that affect sexual function, psychiatric illnesses requiring treatment, and the use of medications known to influence sexual response.

The correlation analysis was conducted with a priori sample size determination. A minimum of 84 participants was required to detect a modest correlation (ρ=0.30) with 80% power and a significance level of α=0.05. The existing sample size of 111 participants for the FSFI was considered adequate.

The Local Ethics Committee approved the study procedure. Informed consent was acquired in writing from all participants. Approved by Sehit Prof Dr Ilhan Varank Sancaktepe Training and Research Hospital, University of Health Sciences Ethics Committee (Approval No: 110, Date: 14/06/2023).

### Sexual function assessment

Female sexual function was assessed using the validated Turkish FSFI questionnaire^2^. The FSFI consists of 19 items evaluating desire, arousal, lubrication, orgasm, satisfaction, and pain. Total scores were calculated according to standard scoring procedures.

### Laboratory measurements

Fasting venous blood samples were collected. Serum homocysteine, vitamin B12, and folic acid levels were measured using standard automated methods. Homocysteine was analyzed as both a continuous variable and a categorical variable using ≤10 and ≤15 μmol/L cut-offs.

### Statistical analysis

Statistical analyses were performed using Statistical Package for the Social Sciences version 26.0 (International Business Machines [IBM] Corp., Armonk, NY, USA). Normality was assessed using the Shapiro-Wilk test. Spearman correlation analyses were used to evaluate associations between biochemical markers and FSFI scores. Group comparisons were performed using an independent-samples t-test or a Mann-Whitney U test as appropriate. Multivariable linear regression analysis was performed with the FSFI total score as the dependent variable and age, homocysteine, vitamin B12, and folic acid as independent variables. Statistical significance was defined as p<0.05.

## RESULTS

A total of 114 sexually active women aged 18–45 years were included in the study. FSFI total and domain scores were available for 111 participants. Serum homocysteine levels were obtained from 99 participants, vitamin B12 levels from 105 participants, and folic acid levels from 103 participants due to incomplete laboratory measurements.

The mean age of the participants was 33.94±7.14 years. The mean FSFI total score was 24.69±4.34 (range: 12.1–36), which is below the commonly accepted cut-off value of 26.55, confirming the presence of sexual dysfunction in the study population. The mean serum homocysteine level was 10.34±2.54 μmol/L (Table 1).

**Table 1:** Demographic and clinical characteristics of the study population.

Variable	n	Mean±SD	Min–max
FSFI desire score	111	3.71±1.05	1.2–6.0
FSFI arousal score	111	3.86±1.00	1.5–6.0
FSFI lubrication score	111	4.43±0.82	2.4–6.0
FSFI orgasm score	111	4.29±0.99	1.6–6.0
FSFI satisfaction score	111	4.51±1.03	1.6–6.0
FSFI pain score	111	3.91±1.49	1.2–6.0
FSFI total score	111	24.69±4.34	12.1–36.0
Age (years)	111	33.94±7.14	18–57
Homocysteine (μmol/L)	99	10.34±2.54	5.2–19.3
Vitamin B12 (pg/mL)	105	317.62±126.68	100–1,046
Folic acid (ng/mL)	103	7.50±3.20	2.1–18.6

Data are presented as mean±standard deviation (SD) and range (min–max). Differences in sample size reflect missing laboratory measurements. SD: standard deviation; FSFI: Female Sexual Function Index.

Spearman correlation analysis demonstrated no significant association between serum homocysteine levels and FSFI total score (ρ=-0.035, p=0.726). Similarly, no significant correlations were observed between FSFI total score and vitamin B12 (ρ=-0.048, p=0.630) or folic acid levels (ρ=-0.093, p=0.350). Age was not significantly correlated with FSFI total score (ρ=-0.150, p=0.120) (Table 2).

**Table 2:** Correlation of Female Sexual Function Index scores with age and biochemical parameters.

Variable		Spearman ρ	p-value
FSFI total score			
	Age	-0.150	0.120
	Homocysteine (μmol/L)	-0.035	0.726
	Vitamin B12 (pg/mL)	-0.048	0.630
	Folic acid (ng/mL)	-0.093	0.350
FSFI desire domain			
	Age	**-0.362**	**<0.001**

Spearman correlation analysis was used. Statistically significant values are shown in bold. FSFI: Female Sexual Function Index.

However, a moderate negative correlation was identified between age and the desire domain of the FSFI (ρ=-0.362, p<0.001), indicating that sexual desire decreased with increasing age.

Participants were categorized according to serum homocysteine levels using a cut-off value of 10 μmol/L. No significant differences were found between groups in FSFI total score (25.31±4.16 vs. 23.95±4.16, p=0.113) or any domain scores. Similar results were observed when using a higher threshold of 15 μmol/L; however, only four participants were in the elevated group, limiting the interpretability of this comparison.

In multivariable linear regression analysis including age, homocysteine, vitamin B12, and folic acid levels as independent variables, the overall model was not statistically significant (R^2^=0.055, p=0.277). None of the variables were identified as independent predictors of FSFI total score (Table 3).

**Table 3:** Multivariable linear regression analysis for Female Sexual Function Index total score.

Variable	B	95%CI	p
Age	-0.12	(-0.28–0.04)	0.13
Homocysteine	-0.03	(-0.21–0.15)	0.72
Vitamin B12	-0.05	(-0.19–0.09)	0.63
Folic acid	-0.08	(-0.25–0.09)	0.35

Model: R^2^=0.055, p=0.277. CI: confidence interval.

## DISCUSSION

In this cross-sectional study of reproductive-age women presenting with sexual dysfunction complaints, no significant association was identified between serum homocysteine levels and overall or domain-specific FSFI scores. Furthermore, homocysteine was not an independent predictor of female sexual function after adjustment for age, vitamin B12, and folic acid levels. These findings suggest that, within this symptomatic clinical population, homocysteine does not appear to play a measurable role in female sexual function.

Our results contrast with a substantial body of evidence in men demonstrating a relationship between elevated homocysteine levels and erectile dysfunction^9-11^. Erectile function is largely dependent on vascular mechanisms, particularly endothelial integrity and nitric oxide–mediated vasodilation. In contrast, female sexual function is inherently multifactorial, involving complex interactions between hormonal, psychological, relational, and vascular components^10-13^. This fundamental physiological difference may partly explain why vascular biomarkers such as homocysteine show a stronger association with male sexual dysfunction than with female sexual function.

Consistent with this interpretation, Yang et al. reported that elevated homocysteine levels were associated with reduced sexual frequency in men but not in women^9^, supporting the concept of sex-specific biological mechanisms. Additional studies have further clarified the vascular role of homocysteine in male sexual dysfunction. For example, Bruk et al. suggested that hyperhomocysteinemia contributes to erectile dysfunction through endothelial injury, oxidative stress, and impaired nitric oxide bioavailability, ultimately reducing penile perfusion^14^. Similarly, Qian et al. demonstrated correlations between serum homocysteine levels and objective penile rigidity parameters measured by AVSS-RigiScan in men^15^. These findings reinforce the importance of vascular pathways in male sexual function, whereas their role in female sexual physiology appears to be less direct and potentially overshadowed by other determinants. The sexual dysfunction is more related to emotional behavior than metabolic dysfunction^16^.

Another important consideration is that most participants in our cohort had homocysteine levels within normal physiological ranges, which may have limited the ability to detect subtle vascular effects. Range restriction in both homocysteine levels and FSFI scores may have further attenuated potential associations.

Age demonstrated a moderate negative correlation with the desire domain but not with the total FSFI score, indicating a domain-specific rather than global effect. This finding is consistent with previous literature suggesting that sexual desire may decline with age independently of other domains of sexual function^5,6^.

We also found no significant associations between vitamin B12 or folic acid levels and sexual function. Given that these parameters were largely within physiological ranges, their variability may not be sufficient to produce clinically meaningful differences in sexual outcomes. This observation aligns with existing evidence emphasizing the multifactorial nature of female sexual dysfunction^4-8^.

Importantly, the absence of a significant association in this study does not exclude the possibility of an association between homocysteine and female sexual function. Rather, our findings indicate that such a relationship was not detectable within this specific clinical sample. The results should therefore be interpreted cautiously, particularly in light of methodological constraints.

### Strengths and limitations

The strengths of this study include the use of a validated multidimensional instrument (FSFI), the assessment of homocysteine using multiple analytical approaches (continuous, categorical, and tertile-based analyses), and the application of multivariable regression analysis.

Several limitations must be acknowledged. First, the cross-sectional design precludes causal inference. Second, the number of participants with markedly elevated homocysteine levels was limited, reducing statistical power for subgroup analyses. Third, important potential confounders such as body mass index, hormonal status, medication use, and psychosocial variables were not systematically assessed. Finally, and most importantly, the study population consisted exclusively of women presenting with sexual dysfunction complaints. This introduces selection and referral bias, restricts variability in FSFI scores, and limits the generalizability of the findings. Therefore, the results should be interpreted within the context of a symptomatic clinical population rather than the general population.

## CONCLUSION

In this cross-sectional study of reproductive-age women presenting with sexual dysfunction complaints, serum homocysteine levels were not associated with overall or domain-specific female sexual function. Homocysteine, vitamin B12, and folic acid were not identified as independent predictors of the FSFI total score.

These findings indicate that, within this symptomatic clinical population, homocysteine does not appear to have a measurable impact on female sexual function. However, given the absence of a healthy control group and the presence of potential confounding factors, the results should be interpreted with caution.

Further large-scale, well-controlled population-based studies incorporating hormonal, metabolic, and psychosocial variables are needed.

## ETHICAL APPROVAL

This study was approved by the Local Ethics Committee with the Approval No. E-4059653-020.

## Data Availability

The datasets generated and/or analyzed during the current study are available from the corresponding author upon reasonable request.
